# Dual-Wavelength On-Chip Integrated Metalens for Epi-Fluorescence Single-Molecule Sensing

**DOI:** 10.3390/s24237781

**Published:** 2024-12-05

**Authors:** Elena Barulina, Dang Du Nguyen, Fedor Shuklin, Mikhail Podobrii, Sergey Novikov, Alexander Chernov, Inki Kim, Aleksandr Barulin

**Affiliations:** 1 Moscow Center for Advanced Studies, Kulakova str. 20, 123592 Moscow, Russia; 2 Department of Biophysics, Institute of Quantum Biophysics, Sungkyunkwan University, Suwon 16419, Republic of Korea; 3 Department of Intelligent Precision Healthcare Convergence, Sungkyunkwan University, Suwon 16419, Republic of Korea; 4 Department of MetaBioHealth, Sungkyunkwan University, Suwon 16419, Republic of Korea

**Keywords:** achromatic metalens, biosensors, photonic integrated circuits, waveguide, fluorescence correlation spectroscopy, single-molecule sensing

## Abstract

Single-molecule fluorescence spectroscopy offers unique capabilities for the low-concentration sensing and probing of molecular dynmics. However, employing such a methodology for versatile sensing and diagnostics under point-of-care demands device miniaturization to lab-on-a-chip size. In this study, we numerically design metalenses with high numerical aperture (NA = 1.1), which are composed of silicon nitride nanostructures deposited on a waveguide and can selectively focus guided light into an aqueous solution at two wavelengths of interest in the spectral range of 500–780 nm. Despite the severe chromatic focal shift in the lateral directions owing to the wavelength-dependent propagation constant in a waveguide, segmented on-chip metalenses provide perfectly overlapping focal volumes that meet the requirements for epi-fluorescence light collection. We demonstrate that the molecule detection efficiencies of metalenses designed for the excitation and emission wavelengths of ATTO 490LS, Alexa 555, and APC-Cy7 tandem fluorophores are sufficient to collect several thousand photons per second per molecule at modest excitation rate constants. Such sensitivity provides reliable diffusion fluorescence correlation spectroscopy analysis of single molecules on a chip to extract their concentration and diffusion properties in the nanomolar range. Achromatic on-chip metalenses open new avenues for developing ultra-compact and sensitive devices for precision medicine and environmental monitoring.

## 1. Introduction

Single-molecule fluorescence is paramount for investigating molecular biophysical processes hidden from ensemble measurements [1]. Its extreme sensitivity and access to temporal dynamic monitoring make it an appealing methodology for sensing biomolecules and diagnostics [2,3,4]. However, optical single-molecule sensing platforms require bulky and expensive elements, which hinder sensing and diagnostics outside the laboratory. Several miniaturized single-molecule platforms have been developed based on 3D-printed microscopes or nanoantenna systems with smartphones [5,6]. Integrating single-molecule sensing units with photonic integrated circuits ultimately results in sensor miniaturization and portability [7]. The development of optical components has undergone a transformative shift with the advent of metasurfaces [8,9,10], leading to the rise of metalenses, that is, flat, ultrathin lenses that manipulate light through engineered nanostructures. Unlike refractive lenses, which rely on curved surfaces to refract light, metalenses modulate the phase in a designated manner, resulting in the desired output field. Metasurfaces modulating the output light phase represent compact optical devices used in imaging, holography, and spectrometry [11,12,13]. Owing to the abundance of low-loss, high-refractive-index materials, metalenses can be designed for a broad range of wavelengths, from ultraviolet and visible [14,15,16] to infrared and terahertz frequencies [17,18]. This versatility presents new possibilities for miniaturized optical systems for various applications ranging from telecommunications to biomedical imaging and sensing [19,20,21]. Their low footprint and complementary metal-oxide semiconductor compatibility make them appealing for creating on-chip optical devices by integrating them into photonic circuits to manipulate light propagation in free space. To control the scattering of light off-chip, meta-atom modes can be coupled to a guided photonic waveguide, inducing modulation of the phase and intensity [22,23]. This enables controlled light emission into free space, such as out-of-plane beam deflection, focusing, and holographic projection [24,25,26,27,28]. This optical device integration unlocks diverse practical applications for data storage, 3D display, and quantum computing [28,29,30].

Despite the increasing interest in integrated metasurface photonics, studies on on-chip metasurfaces for fluorescence-sensing applications remain scarce. To achieve single-molecule sensitivity under epi-fluorescence conditions with state-of-the-art detectors, a high numerical aperture lens with well-overlapped excitation and detection volumes for the wavelengths of interest is required [31]. Free-space metalenses can focus light without chromatic aberrations through phase profile compensation at multiple wavelengths [32,33]. Multilayer-stacked transmissive metalenses unlock additional degrees of freedom to control the phase and amplitude and produce multiwavelength focusing [16,34]. However, multilayer structures on chips require extremely delicate nanofabrication processes. Segmentation of metalens zones represents an efficient way of making multifocal or achromatic transmissive metalenses by modulating the focusing properties of each selected zone [35,36,37]. Recently, single-molecule fluorescence detection was experimentally demonstrated using a free-space transmissive metalens [38]. Unlike transmissive lens optics, the chromatic focal shift produced by an integrated metalens occurs in both the axial and lateral dimensions, making focal volume overlap challenging to achieve. In this study, we demonstrate, to the best of our knowledge, the numerical designs of dual-wavelength metalenses on a planar waveguide with a high numerical aperture (NA = 1.1) that provides perfect focal volume overlap for sets of two separate wavelengths in a large spectral range in the visible and IR regions. The metalenses comprise cylindrical silicon nitride nanoposts on top of a silicon nitride planar waveguide. Furthermore, they are segmented into even rectangular zones designated to vertically focus one of the two wavelengths at a defined focal length. High-NA metalenses provide sufficiently tight focusing to efficiently excite single-point emitters and collect light from them at the design wavelengths. By selecting the metalens design wavelengths according to the favorable excitation and emission wavelengths of the three model fluorophores, ATTO 490LS, Alexa555, and APC-Cy7, we determine the molecule detection efficiencies around the focal volume under epi-fluorescence excitation. Given sufficient collected photons, we simulate fluorescence correlation spectroscopy (FCS) data for molecules diffusing through the metalens detection volume. The possibility of running an FCS represents a unique platform for the single-molecule fluorescence monitoring of chips. Altogether, the epi-fluorescence achromatic metalenses on waveguides pave the way for devices with ultimate miniaturization and sensitivity for portable diagnostic settings and precision medicine.

## 2. Materials and Methods

Numerical simulations of the optical performance of the metalens on the waveguide are performed using the finite-difference time-domain (FDTD) method. The planar waveguide and meta-atoms are composed of silicon nitride [39]. We select silicon nitride as a material for the waveguide and meta-atoms because it exhibits low losses compatible with visible photonic integrated circuitry and an elevated refractive index for effective light coupling. The metalens of size 20 µm is placed on a tapered waveguide. Point spread functions (PSFs) are acquired for three metalens designs that provide achromatic focusing at NA = 1.1 at the excitation and emission wavelengths of the selected model molecules. The fundamental mode TE is injected into the waveguide. The simulation region boundary conditions are perfectly matched layers (PMLs). The maximum collection efficiency of the metalens is estimated by placing an isotropic source in its focal volume and monitoring the power fraction collected inside the single-mode waveguide. The model fluorescent molecules are ATTO 490LS, Alexa Fluor 555, and APC-Cy7, which absorb visible and infrared light. Molecular property data are adopted from the open-source repository [40]. The fluorescent label ATTO 490LS exhibits a large Stokes shift of 165 nm with a maximum absorption peak at 496 nm and a maximum fluorescence peak at 661 nm. APC (Allophycocyanin)-Cy7 is a fluorescent compound with an excitation peak at 651 nm and an emission peak at 779 nm. Alexa Fluor 555 is a fluorescent compound with excitation peaks at 520 and 553 nm and an emission peak at 568 nm. FCS data are simulated via Monte Carlo simulations (SimFCS3 https://www.lfd.uci.edu/globals/ (accessed on 1 September 2024) with 300 molecules diffusing inside a box of 2.4 × 2.4 × 2.4 µm^3^ that is a constituent of cubic unit cells of 50 nm. The molecules move stochastically from one unit cell to another, resembling isotropic 3D diffusion. Autocorrelation functions are reconstructed based on single-molecule brightness and diffusion time.

## 3. Results

**Figure 1:**
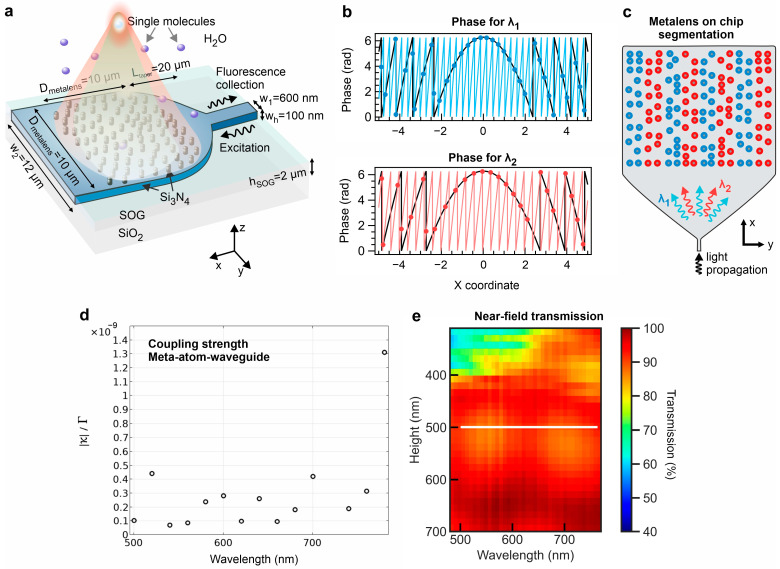
Single-molecule epi-fluorescence sensing by metalens on the chip. (**a**) Scheme of the epi-fluorescence sensing of diffusing single molecules. The metalens dimensions are designated as follows: metalens size (D_metalens_), taper length (L_taper_), narrow single-mode waveguide width (w_1_), wide waveguide width (w_2_), waveguide height (w_h_), thickness of the upper cladding layer of SOG (h_SOG_). The metalens focal length is approximately 7 µm, while the height and diameter of the cylindrical meta-atoms amount to 500 nm and 200 nm, respectively. The taper shape follows a relation: y=α⋅Ltaper2−xm+w22, where α=w1−w22Ltaperm, with *m* being 1.15 and *x* belonging to the range from −*L_taper_*/2 to *L_taper_*/2. (**b**) The meta-atom position produces a phase map focusing on two wavelengths. The phase profiles of the metalenses are represented along the propagation direction of light in the waveguide. (**c**) Segmentation of the metalens into two zones to generate foci at two wavelengths. (**d**) Coupling strength of modes of meta-atom and waveguide in a broad wavelength range. (**e**) Near-field transmission of the meta-atoms. The white line corresponds to the meta-atom height selected for designing integrated metalenses.

Figure 1 shows the design concept, dimensions, and operation of the dual-wavelength metalenses on planar waveguides for single-molecule sensing. The narrow section width (*w*_1_) amounts to 600 nm, and the thickness of the silicon nitride layer is 100 nm. A *V* parameter of a narrow rectangular waveguide can be expressed as V=2πλ·w·nwg2−nclad2 with $w=\sqrt{w_{1}w_{h}}$, *w_h_* denoting the waveguide layer thickness, *n_wg_* being the refractive index of the waveguide material, and *n_clad_* being the refractive index of the silica-on-glass (SOG) cladding layer. The *V* parameter values are below 2.4 at 500 nm or higher wavelengths, which meets the condition of a single-mode waveguide. The excitation wavelength is focused by the metalens inside a water medium with a high NA to provide efficient excitation of freely diffusing molecules and a large collection efficiency (Figure 1a). However, to support substantial fluorescence collection, the emission light around the maximum of fluorophores has to be collected exactly from the excitation focal volume [31,38]. To provide detection only from the focal volume, we add a cladding layer of silica-on-glass (SOG) a few micrometers thick to prevent evanescent waves of the guided mode from reaching the water medium. We segment a metalens into equal alternating rectangular zones of two types, where one type is designed to produce a focal volume at the excitation wavelength (*λ*_1_), and the other type generates a focal volume at the same place at a substantially different emission wavelength (*λ*_2_). To mimic the phase pattern of the outcoupled light in free space, we assess the phase accumulation inside the waveguide. We numerically simulate a periodic diffraction grating of cylindrical nanoposts on top of a waveguide. By monitoring the diffraction angle in the far field, we can deduce the propagation constant (β) according to the following relation: βλ=2πnλsinθ+2πΛ , with *n* denoting the refractive index of the top cladding, *θ* the angle of the outcoupled radiation, and *Λ* the grating period. By setting *Λ* at 365 nm, the grating supports close-to-vertical light propagation at a large frequency band in the visible spectrum. Then, we set the meta-atoms in the intersection positions of the waveguide-accumulating phase ($\varphi_{wg}$) and the lens phase equation ($\varphi_{lens}$). Moreover, to correct the curved wavefront after light propagation through the taper, we compensate for the input phase $\varphi_{input}(\lambda )$ passing by the first meta-atoms. Thus, the meta-atom positions *(x,y)* satisfy the condition $\varphi_{wg}(x,y)\approx \varphi_{lens}(x,y)$ (Figure 1b). These two functions are expressed as follows:

(1) $$\varphi_{wg}\left(x,y\right)=\sum_{i=1}^{2}[\beta \left(\lambda_{i}\right)\cdot x+\varphi_{input}(y,\lambda_{i})]{Loc}_{i}\left(y\right),$$

(2) $$\varphi_{lens}\left(x,y,\lambda \right)=\sum_{i=1}^{2}\frac{2\pi n}{\lambda_{i}}\left(F-\sqrt{F^{2}+x^{2}+y^{2}}\right){Loc}_{i}\left(y\right).$$

Here, ${Loc}_{i}\left(y\right)$ equals zero for *y* values outside the *i*th segment and one for *y* values inside the *i*th segment (Figure 1c), and *F* is the focal distance of the metalens on the waveguide in the cladding medium.

We select *F* according to the design numerical aperture as: NA=n·sin⁡(arctan⁡D2F). The focal distance for such a metalens size exceeds ten wavelengths for the selected excitation wavelengths, making it a purely far-field-based detection system. Equation (1) for $\varphi_{wg}$ is valid as long as the coupling strength between a meta-atom and waveguide remains weak to ensure that the coupling strength κ is significantly lower than the imaginary part Γ of the eigenfrequency Ω−iΓ of the hybrid cylinder-waveguide mode. The coupling strength is defined via the coupled modes theory with the integral $\kappa \propto \int_{\Sigma }d\Sigma \;(E_{wg}^{*}\times H_{cyl}+E_{cyl}\times H_{wg}^{*})$ over the base of the cylinder Σ (being the scattering port) of the fields $E_{cyl}$ and $H_{cyl}$ of the quasi-normal mode of the cylinder and fields $E_{wg}$ and $H_{wg}$ of the guided wave [41]. Note that this approach would work for TE polarization better since the scattering for this polarization is simpler and waveguide-cylinder scattering is weaker, which is one of the main reasons we consider a fundamental TE mode. To avoid normalization difficulties of radiative quasi-normal modes, one can consider the ratio of coupling strength to Γ (Figure 1d). Our calculations show that this ratio does not exceed $10^{-9}$, proving that the waveguide and cylinder modes are weakly coupled. The coupling strength rises with the fill factor of the junction area of the meta-atom and the waveguide. The meta-atom diameter is fixed at 200 nm to provide a sufficiently high coupling strength without perturbing the phase accumulation process in the waveguide. An increase in the diameter leads to perturbed focusing in free space and a low inter-cylinder distance, complicating the device fabrication process. By propagating a plane wave from the waveguide through the meta-atoms we evaluate the near-field transmission. The transmission is reduced at low meta-atom heights (Figure 1e); therefore, the selected height of 500 nm yields a transmission of approximately 90% over a broad wavelength range. The high transmission values are within the reach for numerically optimized heights thanks to the nearly lossless nature of silicon nitride in the visible spectrum.

**Figure 2:**
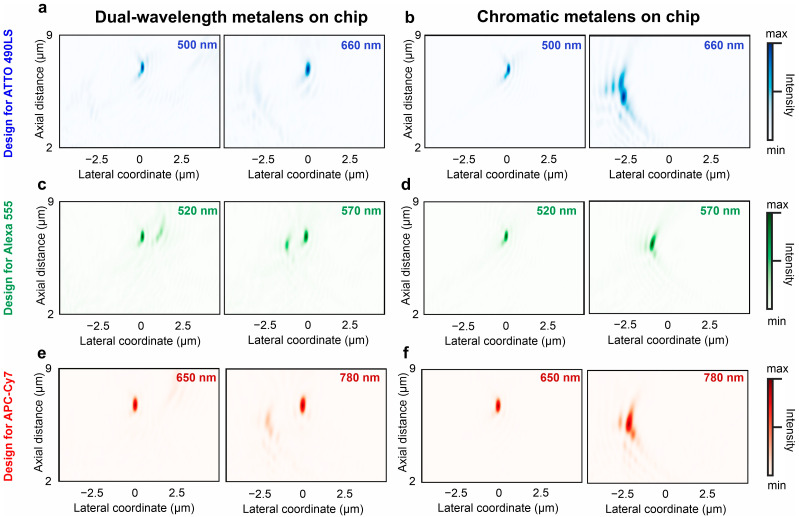
Focal point overlap comparison of the dual-wavelength and chromatic metalenses on the waveguide. (**a**) Point spread function (PSF) of segmented metalens for ATTO 490LS fluorescence detection at 500 and 660 nm. (**b**) PSF of single-wavelength metalens at 500 and 660 nm. (**c**) PSF of segmented metalens for Alexa 555 fluorescence detection at 520 and 570 nm. (**d**) PSF of single-wavelength metalens at 520 and 570 nm. (**e**) PSF of segmented metalens for APC-Cy7 fluorescence detection at 650 and 780 nm. (**f**) PSF of single-wavelength metalens at 650 and 780 nm. The color bars depict optical field intensity.

To confirm the potential of this methodology for single-molecule sensing on a chip, we select three fluorophores with large and modest Stokes shifts as model analytes: ATTO 490 LS, Alexa Fluor 555, and APC-Cy7 tandem molecules. These models rely on molecular spectrum, quantum yield, and fluorescence lifetime datasets. Thus, three dual-wavelength metalens designs are modeled to efficiently excite the molecules and collect fluorescence around the emission maximum as follows: 500 and 660 nm for ATTO 490LS, 520 and 570 nm for Alexa 555, and 650 and 780 nm for APC-Cy7. Light propagation from the single-mode waveguide toward the metalens on top of the taper and coupling to free-space radiation is simulated using FDTD. We retrieve the PSFs for the three designs at the wavelengths of interest. The dual-wavelength metalenses on the chip produce well-defined focal spots in a water medium with well-overlapping contours along the axial and lateral directions (Figure 2a,c,e). For comparison, we also simulate a one-segment metalens on the chip for the excitation wavelength only (Figure 2b,d,f). The focal spots at the emission wavelengths are formed at highly deviated positions in both the axial and lateral directions. Although the axial position shift arises from the wavelength-dependent lens phase profile, the difference in the propagation constant produces a change in the propagation angle, which leads to a drastic lateral focal position shift and focal volume distortion. This significant focal volume mismatch makes epi-fluorescence sensing with chromatic integrated metalenses elusive. In the case of dual-wavelength metalenses, the large wavelength separation almost completely prevents the second-focus residual intensity in the PSF (Figure 2a,e). However, the residual intensity from the second focus remains minor even for Stokes shifts of 50 nm (Figure 2c) because light propagation along the vertical direction from the metalens remains more favorable. The focusing efficiency of the metalens varies from 8% to 10% for each wavelength, which competes well with the independent designs of single-wavelength integrated metalenses [42]. Owing to the symmetry of the scattering matrix of the metalens–waveguide–environment system, the dynamics of such a system is reciprocal [43]. Therefore, the problem of light outcoupling from the waveguide and incident light collection by the waveguide from free space can be considered equivalent.

**Figure 3:**
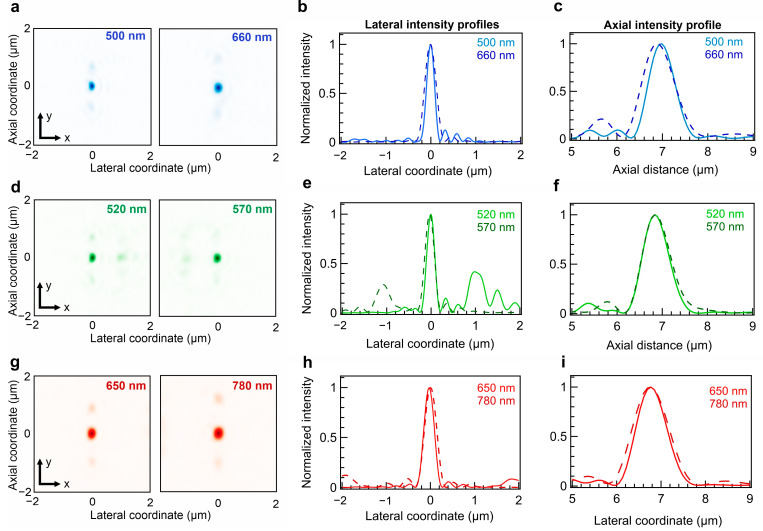
Lateral PSF and linear intensity profiles of dual-wavelength metalenses on the chip. (**a**) Lateral PSF of metalens for ATTO 490LS fluorescence detection at 500 and 660 nm. (**b**) Horizontal cut of the images in (**a**). (**c**) Axial intensity profiles of the metalens for ATTO 490LS fluorescence detection at 500 and 660 nm. (**d**) Lateral PSF of metalens for Alexa 555 fluorescence detection at 520 and 570 nm. (**e**) Horizontal cut of the images in (**d**). (**f**) Axial intensity profiles of the metalens for Alexa 555 fluorescence detection at 520 and 570 nm. (**g**) Lateral PSF of metalens for APC-Cy7 fluorescence detection at 650 and 780 nm. (**h**) Horizontal cutoff of the images in (**g**). (**i**) Axial intensity profiles of the metalens for APC-Cy7 fluorescence detection at 650 and 780 nm.

In the context of FCS produced with an epi-fluorescence configuration, diffusing molecule emissions can be detected only if the excitation and detection focal volumes overlap. The metalens focal spots at molecule excitation and emission wavelengths exhibit a perfect spatial match in the lateral dimensions, proving that the difference of *φ_wg_* at two wavelengths is compensated for producing focusing with high NA (Figure 3a,d,g). The beam diameter full width at half maximum (FWHM_x/y_) approaches the diffraction limit of conventional high-NA objective lenses. The metalenses designed for the Alexa 490 LS, Alexa 555, and APC-Cy7 tandem molecules have beam diameters of 220, 250, and 276 nm, respectively, at the corresponding excitation wavelengths (Figure 3b,e,h). The beam diameters approach the diffraction-limited performance of analog free-space lenses. Similarly, the axial chromatic shift is corrected for all three designs, and the focal distance mismatch is <50 nm (Figure 3c,f,i).

**Figure 4:**
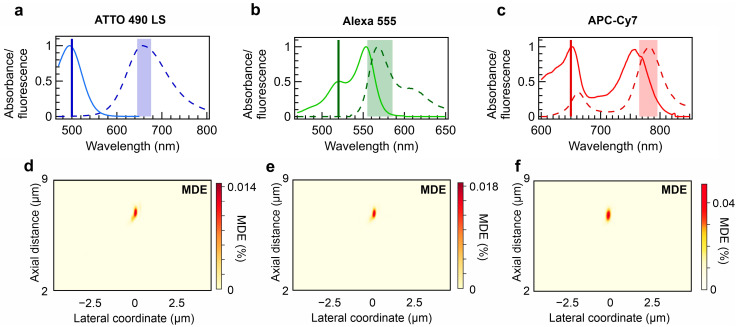
Molecule detection efficiency under epi-fluorescence configuration. Absorbance and fluorescence spectra of (**a**) ATTO 490 LS, (**b**) Alexa 555, and (**c**) APC-Cy7. The spectral data are adopted from [40]. The solid line spectra denote absorbance, whereas the dashed line spectra refer to emission. In addition, the solid lines denote the design excitation wavelength, and the shading indicates the simulated collection band by the metalens. The spectra-computed molecule detection efficiency with dual-wavelength metalenses designed for (**d**) ATTO 490 LS, (**e**) Alexa 555, and (**f**) APC-Cy7.

In this case, the molecular detection efficiency (*MDE*) in the epi-fluorescence configuration is represented as follows [44,45]:

(3) $$MDE\left(x,y,z\right)=I_{exc}(x,y,z)\cdot CEF(x,y,z),$$

where *I_exc_* is the excitation intensity profile, and *CEF* is the emission collection efficiency within a detection frequency band. The molecular dipole rotation processes occur several orders of magnitude faster than the diffusion through a microscopic focal spot; therefore, diffusing molecules are typically considered isotropic point sources in the context of 3D diffusion FCS. The collection efficiency of the isotropic diffusion source in the focal volume is expressed as follows:

(4) $$CEF\left(x,y,z\right)=P_{max}\frac{\int F\left(\lambda \right)\cdot I_{det}(x,y,z,\lambda )d\lambda }{\int F\left(\lambda \right)d\lambda },$$

with *P_max_* denoting the fraction of coupled emitted photons into the waveguide from an isotropic light source located in the focal volume at the emission maximum *I_det_* PSFs at the detection band around the emission wavelength. *F(λ)* denotes the fluorescence intensity spectra of the molecules of interest (Figure 4a–c). Because the metalens design operates in the *TE*_0_ mode, only one-third of the photons emitted by the isotropic source can be collected. The proposed metalens designs yield MDE values of 0.015–0.05% for ATTO 490LS, Alexa 555, and APC-Cy7 (Figure 4d–f). Regardless of this low value, even objective lenses of high transmission and aberration correction quality notably often yield MDE values between 0.1% and 1% [46]. In contrast, the miniaturized on-chip metalens exhibits an extremely small thickness of 500 nm and a lateral size of 20 µm. They can be produced in a scalable manner within photonic integrated circuits.

**Figure 5:**
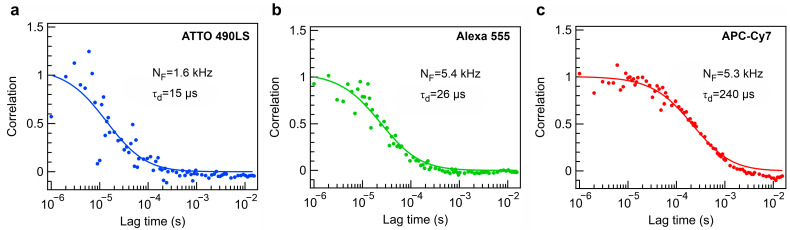
Metalens-based diffusion autocorrelation functions for molecules of interest. Simulated FCS data with fitting curves are represented for (**a**) ATTO 490 LS, (**b**) Alexa 555, and (**c**) APC-Cy7. The fit functions correspond to a 3D diffusion FCS model.

To numerically assess the possibility of collecting a sufficient number of photons from single diffusing molecules, we utilize the formalism of molecular emission based on a two-level system with a low excitation intensity. The number of collected photons from the molecules in the absence of photobleaching is determined as $N_{F}=MDE\cdot \psi \cdot \bar{S_{1}}\cdot k_{fl}$ [47,48], where *ψ* denotes the quantum yield of the molecule. $\bar{S_{1}}$ denotes the time-independent average population of the singlet excited state. In addition, it amounts to $k_{ex}/(k_{fl}+k_{ex})$, with $k_{fl}$ being the fluorescence rate constant and *k_ex_* being the excitation rate constant. By exploiting available datasets of quantum yields, fluorescence spectra, and fluorescence lifetimes ($\tau_{fl}=1/k_{fl}$) for the molecules of interest, we compute the number of collected photons per molecule under the condition of $k_{ex}=0.1k_{fl}$, which would support a negligible photobleaching probability condition in real experimental applications. We estimate that our metalens designs enable single-molecule count rates of 1.6, 5.4, and 5.3 kcounts/s for ATTO 490LS, Alexa 555, and APC-Cy7 molecules, respectively. These single-molecule brightness levels exceed the requirements for diffusion FCS, which allows direct probing of the number of molecules and their size based on fluorescence intensity fluctuations (Figure 5a–c). The possibility of observing autocorrelation functions within a reasonable acquisition time would serve as robust proof of single-molecule sensitivity [49] and a read-out of the biomolecule size and concentration. The acquisition time set in the simulations is 30 s, which complies with the standard FCS sensing measurement settings. The fluorescence correlation functions are reconstructed with a high signal-to-noise ratio (SNR), whereas the latter increases with higher *N_F_* and MDE, as expected from theory. The autocorrelation function noise decreases linearly with an increase in *N_F_*. Although other loss channels may lead to reduced *N_F_*, they can be partly compensated by *k_ex_* or accumulation time increase. The detection volume of the metalens in the far field is approximately 0.17 fl, which can be applied to studies on molecular dynamics at nanomolar concentrations.

## 4. Discussion

To the best of our knowledge, this is the first numerical demonstration of a dual-wavelength metalens on a planar waveguide with a high numerical aperture. Photonic integrated circuits represent the ultimate miniaturization scheme for optical sensing devices [7], making this methodology valuable for truly portable sensors. The proposed metalens design relies on the spatial segmentation of meta-atom regions, where each segment produces vertical focusing of light at one or the other wavelength. The design can be easily tuned by choosing the two wavelengths over a broad spectral range. By selecting wavelengths for favorable molecule fluorescence excitation and collection, we model the epi-fluorescence sensing of diffusing single molecules of ATTO 490 LS, Alexa 555, and APC-Cy7. Owing to the extreme focal shift in the lateral and axial directions for substantial wavelength separation, the proposed method excites and collects single-molecule fluorescence on a chip for molecules with large Stokes shifts. Owing to the perfect overlap of the focal volumes and the high NA of the metalens, the count rate per single molecule reaches a few thousand photons per second. This sensitivity level is sufficient for FCS to probe the concentration, size, and molecular interactions, or the binding of a labeled biomarker at nanomolar concentrations on a chip. The proposed methodology can be extended to multi-wavelength spectroscopy; however, the drawbacks of reducing focusing efficiency and complication of the multifocal pattern may yield to the performance of dual-wavelength operation for epi-fluorescence single-molecule sensing. Although the proposed methodology requires state-of-the-art fabrication facilities and complex optical setups for experimental concept demonstration, the geometries of the silicon nitride nanoposts and tapered waveguides can be treated with state-of-the-art nanofabrication technologies. Similar to near-field metasurfaces that allow sensitive biomarker detection, far-field on-chip metalenses can sense low analyte concentrations [50,51,52] while probing the exact concentration, molecular size, and possible intermolecular interactions. We believe that this platform will open prospective routes toward broad applications of miniaturized integrated metalenses as portable single-molecule sensors for diagnostics, precision medicine, and environmental monitoring.

## Data Availability

Data are contained within the article.
